# Analysis of scientific production on the new coronavirus (COVID-19): a bibliometric analysis

**DOI:** 10.1590/1516-3180.2020.0449.R1.01102020

**Published:** 2021-01-15

**Authors:** Erika Morganna Neves de Oliveira, Ana Raquel Batista de Carvalho, Joyce Soares e Silva, Antônio Rosa de Sousa, Maria Eliete Batista Moura, Daniela Reis Joaquim de Freitas

**Affiliations:** I Doctoral Student. Postgraduate Nursing Program, Universidade Federal do Piauí (UFPI), Teresina (PI), Brazil.; II Master’s Student. Postgraduate Nursing Program, Universidade Federal do Piauí (UFPI), Teresina (PI), Brazil.; III Master’s Student. Postgraduate Nursing Program, Universidade Federal do Piauí (UFPI), Teresina (PI), Brazil.; IV Nursing Student. Nursing Department, Universidade Federal do Piauí (UFPI), Teresina (PI), Brazil.; V PhD. Professor and Postgraduate Nursing Program, Universidade Federal do Piauí (UFPI), Teresina (PI), Brazil.; VI PhD. Professor, Department of Microbiology, Universidade Federal do Piauí (UFPI), Teresina (PI), Brazil.

**Keywords:** Coronavirus infections, Nursing research, COVID-19 [supplementary concept], Researchers, Bibliometric study, Bibliometric indicators, Scientific and technological activities.

## Abstract

**BACKGROUND:**

The pandemic of the new coronavirus has culminated in a scientific race to seek knowledge about this virus and its treatments, vaccines and preventive strategies, in order to reduce its impact on healthcare and economics worldwide. Hence, it is important to recognize the efforts of researchers who are at the forefront of investigations relating to the new coronavirus.

**OBJECTIVE:**

The present study was carried out with the aim of analyzing the world scientific production relating to COVID-19.

**DESIGN AND SETTING:**

Exploratory and descriptive bibliometric study conducted in the city of Teresina (PI), Brazil.

**METHOD:**

ISI Web of Knowledge/Web of Science (WOS) was chosen as the database. Data-gathering was carried out in May 2020. The data analysis was performed using the HistCite^TM^ software, version 9.8.24, and the VOSviewer bibliometric analysis software, version 1.6.8.

**RESULTS:**

2,625 published papers that included descriptors within the scope of this investigation were identified. These articles were published in 859 different journals that are indexed in WOS, by 9,791 authors who were linked to 3,365 research institutions, located in 105 countries.

**CONCLUSION:**

Ascertaining scientific production through a bibliometric analysis is important in order to guide researchers on what has already been produced and what is being researched, so as to be able to address gaps in knowledge through future research.

## INTRODUCTION

On December 31, 2019, the World Health Organization (WHO) reported the first outbreak of pneumonia in Wuhan City, Hubei Province, China. It was discovered shortly afterwards that this pneumonia was due to a new coronavirus, with genetic characteristics, mode of infection and hosts distinct from the other coronaviruses that were already known. It was given the scientific name of severe acute respiratory syndrome coronavirus 2 (SARS-CoV-2) and the infection (disease) that it causes was named COVID-19 (coronavirus disease 2019).^1^^,^^2^ At the end of January 2020, this epidemic outbreak turned into a pandemic and thus into a public health emergency of international interest.^3^

According to information from the Pan American Health Organization, the pandemic had affected more than 180 countries as of June 30, 2020, with confirmation of 10,185,374 cases of COVID-19 worldwide and 503,862 deaths from it.

 Since the beginning of the pandemic, governments and scientists have been working on solutions to prevent rapid spread of the virus and contagion. Healthcare organizations have coordinated a series of protocols and guidelines aimed at improving rapid circulation of information about the pathology and possible treatment protocols and thereby mitigating the impact of the disease. However, despite the research carried out, the transmission mechanisms and clinical spectrum of the disease are still not fully understood, and there is still a lack of treatments and vaccines to control COVID-19.^2^^,^^4^^,^^5^

In view of this problem, it is essential that scientific production of studies on COVID-19 should be analyzed and expanded. Bibliometric studies have the aim of investigating the collaborative and scientific production network on a research topic, which in this case is on the new coronavirus. This knowledge facilitates recognition of researchers who produce and publish the most on the topic.

## OBJECTIVE

The questions that guided this study were the following: Which information sources are of value regarding COVID-19, through the metrics of authorship and citation? What analysis has been done on the indicators of the dynamics and evolution of scientific and technological information about COVID-19? Thus, in the light of these questions, the objective of this study was to analyze the worldwide scientific production relating to COVID-19.

## METHODS

### Research design

This was an exploratory and descriptive bibliometric study with a quantitative approach that was conducted through defining a database for consultation and the criteria to be used in data-gathering and data representation and analysis.^6^

### Data-gathering period

Data-gathering was carried out in May 2020. Search periods available in the database for complete years (1945-2020) were used, in order to allow replication or updating of this study without the need to conduct it again from its inception. Because COVID-19 is a recent topic, the search found that the first result was published in 2019, the year in which the first case of COVID-19 was registered. For this reason, the time period evaluated was from December 2019 to May 11, 2020.

### Selection criteria

No refinement filters relating to fields of knowledge, countries or languages of the studies were used. All records of published studies in which the scope of the study included descriptors relating to the research topic were covered.

### Data-gathering

The steps followed three procedures: defining the database to be consulted; determining the criteria to be used for data-gathering; and defining the representation and analysis of the data gathered.

ISI Web of Knowledge/Web of Science (WOS) was chosen as the database because of its “academic recognition of being considered one of the most comprehensive bases in several areas of scientific knowledge” and because this database has an important position as a pioneer in bringing together journals from more than 100 fields of knowledge.^7^

The descriptors were defined from the Medical Subject Headings (MeSH) catalogue, from which the following search terms were selected: “(COVID-19) OR (“2019 novel coronavirus disease”) OR (“COVID19”) OR (“COVID-19 pandemic”) OR (“SARS-CoV-2 infection”) OR (“COVID-19 virus disease”) OR (“2019 novel coronavirus infection”) OR (“2019-nCoV infection”) OR (“coronavirus disease 2019”) OR (“coronavirus disease-19”) OR (“2019-nCoV disease”) OR (“COVID-19 virus infection”)”. The quotation marks indicate the exact representation of terms with more than one word. Data-gathering was carried out by searching for these terms, which represented article titles, abstracts, authors’ keywords and created keywords.

In this manner, 2,625 articles were identified, and these were used as a set of articles for the bibliometric analyses proposed. It should be noted that all articles found were selected for this investigation, since the focus of the study was to ascertain all the production that had occurred up to the end of the data-gathering period.

### Data processing and analysis

The data gathered were then analyzed by exporting these data to the HistCite^TM^ software, version 9.8.24. HistCite is a software package used for bibliometric analysis and information visualization. It was developed by Eugene Garfield, founder of the Institute for Scientific Information and inventor of important information retrieval tools such as Current Contents and the Science Citation Index. This package was used to organize the information and facilitate the analysis. The following items were analyzed: journals with the highest number of records and the number of articles distributed according to the country of origin of the authors.

In addition to these data generated through the software, aspects of the ten articles most cited across the entire WOS were elucidated in order to identify their main contributions to the topic of COVID-19. In addition, an analysis on indicators of the dynamics and evolution of scientific and technological information on this topic was carried out.

The VOSviewer software, version 1.6.8, was used to analyze co-competition networks between keywords. VOSviewer (Visualization of Similarities Viewer) is part of a free software suite for bibliometric analysis and visualization. It was developed by Van Eck and Waltman and is available at: www.vosviewer.com. In analyzing these co-competition networks, it was possible to map out possible research topics relating to COVID-19. The sizes of the nodes that were produced in the networks indicated the frequency of occurrence of keywords, and the relationships between nodes became stronger as the proximity between them became greater.

### Ethical aspects

Since this was a bibliometric study, it was not necessary to submit the research project to an ethics committee for research on humans. According to Resolution 466 of 2012, of the National Health Council of Brazil, there is no need for approval from a research ethics committee for studies that use secondary data. However, the present researchers are committed to maintaining the ethical principles recommended for research of this nature, through respecting ideas and citations and referencing authors and their publications.

## RESULTS

In this investigation, 2600 published articles that included descriptors within the scope of this topic were identified. The articles were published in 859 different journals indexed in the WOS, by 9,791 authors who were linked to 3,365 research institutions, located in 105 countries. To produce these articles, 25,053 references were used, with an average of 10 references per article.

China was the country with the largest number of published articles, presenting 645 records. The United States, United Kingdom and Italy were next, with 595, 270 and 269 records, respectively. Brazil was in 15^th^ position, with 48 published papers registered up to the time of the search in this database. The list of the first 15 countries with the largest numbers of articles published in WOS can be seen in Table 1.

**Table 1.** List of countries with the most production on the topic of COVID-19 in the Web of Science (WOS) database

Ranking	Countries	Number of scientific articles published
1^st^	China	645
2^nd^	United States	595
3^rd^	United Kingdom	270
4^th^	Italy	262
5^th^	India	136
6^th^	Germany	112
7^th^	Iran	107
8^th^	Australia	96
9^th^	Switzerland	86
10^th^	France	77
11^th^	Singapore	56
12^th^	South Korea	55
13^th^	Turkey	49
14^th^	Spain	49
15^th^	Brazil	48

Source: based on data from the Web of Science.

The journals with the highest numbers of published articles were The Lancet, which had 1,602 citations, and the Journal of Medical Virology, which had 199 citations, from 84 and 69 published papers, respectively. To identify journals with high citation impact, an index was defined by dividing the number of citations by the number of published studies.

The list of the ten journals with the most relevant scientific productions on the subject of COVID-19 is shown in Table 2.

**Table 2.** List of journals with the most production on the topic of COVID-19 in the Web of Science (WOS) database

Ranking	Journal	Country	Number of citations	Number of published scientific articles	Citation index
1^st^	The Lancet	United Kingdom	1,602	84	19.1
2^nd^	Journal of Medical Virology	United States	199	69	2.9
3^rd^	British Medical Journal (BMJ)	United Kingdom	170	215	0.8
4^th^	Canadian Journal of Anesthesia − Journal Canadien d’Anesthésie	Canada	69	21	3.3
5^th^	Eurosurveillance	France	44	28	1.5
6^th^	Cureus	United States	18	54	0.3
7^th^	Head and Neck − Journal for the Sciences and Specialties of the Head and Neck	United States	10	38	0.2
8^th^	Indian Journal of Community Health	India	0	24	0.0
9^th^	Archives of Bond and Joint Surgery (ABJS)	France	0	23	0.0
10^th^	Archives of Academic Emergency Medicine	Iran	6	21	0.3

Source: based on data from the Web of Science.

The ten most cited authors relating to the topic of COVID-19 are shown in Table 3 according to author, title, journal and number of citations, which ranged from 74 to 623.^8^^,^^9^^,^^10^^,^^11^^,^^12^^,^^13^^,^^14^^,^^15^^,^^16^^,^^17^

**Table 3.** List of the most cited scientific articles relating to the topic of COVID-19 in the Web of Science (WOS) database

Ranking	Author	Title	Journal	Number of Citations
1^st^	Huang et al.^8^	Clinical features of patients infected with 2019 novel coronavirus in Wuhan, China	The Lancet	623
2^nd^	Chen et al.^9^	Epidemiological and clinical characteristics of 99 cases of 2019 novel coronavirus pneumonia in Wuhan, China: a descriptive study	The Lancet	352
3^rd^	Lu et al.^10^	Genomic characterisation and epidemiology of 2019 novel coronavirus: implications for virus origins and receptor binding	The Lancet	211
4^th^	Holshue et al.^11^	First Case of 2019 Novel Coronavirus in the United States	New England Journal of Medicine	156
5^th^	Wu et al.^12^	A new coronavirus associated with human respiratory disease in China	JAMA	139
6^th^	Rothe et al.^13^	Transmission of 2019-nCoV Infection from an Asymptomatic Contact in Germany	New England Journal of Medicine	116
7^th^	Zhou et al.^14^	Clinical course and risk factors for mortality of adult inpatients with COVID-19 in Wuhan, China: a retrospective cohort study	The Lancet	101
8^th^	Zou et al.^15^	SARS-CoV-2 Viral Load in Upper Respiratory Specimens of Infected Patients	New England Journal of Medicine	79
9^th^	Chen et al.^16^	Clinical characteristics and intrauterine vertical transmission potential of COVID-19 infection in nine pregnant women: a retrospective review of medical records	The Lancet	76
10^th^	Liang et al.^17^	Cancer patients in SARS-CoV-2 infection: a nationwide analysis in China	Lancet Oncology	74

Source: based on data from the Web of Science.

**Figure 1.**
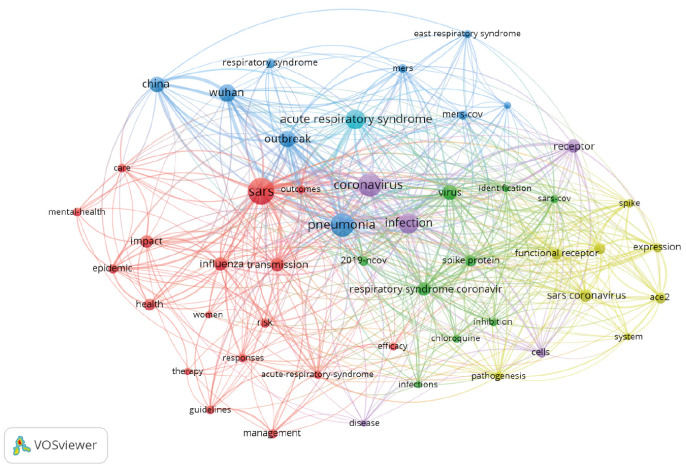
Co-occurrence networks of keywords relating to the topic of COVID-19 in the Web of Science (WOS) database.

Source: based on data from the Web of Science.

Figure 1 shows the keyword co-occurrence networks for the 2,625 documents in the sample. To facilitate visualization, construction of the network was restricted to keywords with ten or more occurrences, which resulted in 49 nodes that were organized into six different colors, namely: blue, red, green, lilac, yellow and turquoise (clusters). These were the words that most frequently determined the central theme of a body of documents.

## DISCUSSION

The COVID-19 pandemic had led to publication of a large number of scientific studies on this subject, conducted around the world. The global dimensions of the direct and indirect effects of the coronavirus have required quick responses, which have placed scientific production and dissemination at the center of attention. Thus, the bibliometric analysis carried out through this study have enabled characterization of these researchers during the pandemic. ^8^^,^^9^^,^^10^^,^^11^^,^^12^^,^^13^^,^^14^^,^^15^^,^^16^^,^^17^

China has contributed the largest number of scientific published papers during the COVID-19 pandemic, according to the analysis carried out here (Table 1). This can be explained by the fact that China is home to more than 3.61 million licensed doctors, and that this country was the cradle of the current pandemic.^17^ The United States is second in the ranking. This can be explained by the fact that it accounts for the largest number of scientific journals on the search platforms used, in addition to being a country in which researchers around the world are interested in publishing their results.

In addition, a large number of authors have taken the opportunity presented by the pandemic and the importance of this topic to increase their numbers of published studies through submitting studies in the form of letters to the editor and short communications. They have filled an urgent need to build up evidence for clinical practice and for organization of healthcare services and systems and intersectoral coping actions.

It can be supposed that the countries that had the next largest numbers of major productions, including the United Kingdom, Italy, India and Germany, were in this position because the first phase of the pandemic reached them shortly after China announced the epidemic outbreaks in that country. These countries also have research laboratories with more robust funding for searching for treatments against the virus. In the case of Brazil, the effects of the disease began to appear later and, thus, this country’s scientific production is still rising.^18^^,^^19^^,^^20^^,^^21^^,^^22^

Regarding the journals with the highest numbers of citations in WOS, The Lancet, Journal of Medical Virology and British Medical Journal (BMJ) occupied the first, second and third positions, respectively. This was because they are journals of global scope, with high impact factors and rapid publication, and with open calls for submission of manuscripts on this topic.^18^^,^^19^^,^^20^^,^^21^^,^^22^

With 623 citations in WOS, the published paper with the greatest impact was by Huang et al.,^8^ which was published in The Lancet. This article aimed to describe the epidemiology and the clinical, laboratory and radiological characteristics of patients with COVID-19 infection and to compare characteristics between intensive care and non-intensive care patients. This study was carried out shortly after the discovery of the virus and, thus, the authors showed that gaps existed with regard to knowledge of the origin, epidemiology, duration of human transmission and clinical spectrum of the disease.

Another factor observed was co-occurrence of relationships between pairs of keywords that were determined from the numbers of articles in the database that occurred together, whether in the title, in the abstract or in the list of keywords.^23^^-^^24^

In analyzing these networks, it was possible to map out possible research topics on COVID-19. The size of the node indicated the frequency of occurrence of a keyword, and the closer together they were, the stronger their relationship was.

Cluster 1 (red) relates to research addressing the epidemiological picture of the virus since its identification in December 2019. The second cluster (green) shows that, so far, no proven effective treatment for COVID-19 has been found and, hence, researchers have been working on the search for the best therapy for coping with the new coronavirus. The words cited in this cluster suggest that this type of approach was used in the studies analyzed.

Among the nine nodes grouped in cluster 3 (blue), there is a research trail addressing the origin of the disease in Wuhan, in Hubei province, China, where the third coronavirus outbreak in human history occurred. This group also addresses the Middle East respiratory syndrome coronavirus (MERS-CoV), which has a zoonotic origin and is associated with severe and potentially fatal respiratory failure. It is noteworthy that MERS-CoV was the agent responsible for the outbreak in the Middle East that originated in Saudi Arabia, in 2012, with 2,494 cases recorded in 27 countries and 858 deaths (34% lethality).

The fourth cluster (yellow) relates to the behavior of the molecular structures of COVID-19. The nucleic acid of the new coronavirus is a positive-stranded ribonucleic acid (RNA), and its structural proteins include the following: spike protein (S), envelope protein (E), membrane protein (M) and nucleocapsid phosphoprotein. It has been shown that the new coronavirus enters epithelial cells through the spike protein and interacts with the host’s angiotensin-converting enzyme 2 (ACE2) receptor protein on the surface, thus causing human infection.

The fifth cluster (lilac) reveals the researchers’ interest in describing the performance of viral cells in the host organism. These cause serious infection due to the inability to exchange carbon dioxide and oxygen between lung cells, thereby causing breathing difficulties.

The sixth cluster (turquoise) shows that the most severe cases of COVID-19 evolve into respiratory distress syndrome, which is the main cause of hospitalization and requires immediate care in an intensive care unit.

The findings from this investigation show that bibliometric studies have the important function of characterizing the research carried out on the topic in Brazil and around the world. Through this research design, the origins, institutions, researchers and number of citations of scientific production are noted. In addition, the segments within the topic of COVID-19 that have been most studied can be discerned.

However, the present bibliometric study has limitations. Only a single database was used, i.e. Web of Science^TM^. Although this is a referential platform for scientific citations that was designed to support scientific and academic research with wide coverage in the fields of science and social sciences, it may be necessary to deepen the search using other scientific databases, through further studies. The high number of studies indexed in this database every day made it impossible to analyze them daily, and this can also be cited as a limitation. This led the present researchers to choose to delimit a period within which to obtain data, in order to be able to proceed with their investigation and discussion. Thus, some information may have been lost in this process and the reality may not match the data gathered in the present study.

## CONCLUSION

The sources of value regarding COVID-19, which were recognized by means of authorship and citation metrics, comprised 10 studies, among 623 papers published in 859 different journals indexed in the Web of Science, written by 9,791 authors who have links with 3,365 research institutions, located in 105 countries.

The analysis on the indicators of the dynamics and evolution of scientific and technological information on COVID-19 showed that there are gaps in the knowledge of this subject. These gaps are wide and diversified. No join-ups between studies, authors and institutions around the world were demonstrated. There is a need to build knowledge networks within this field that enable more studies that are capable of contributing to the improvement of scientific evidence relating to coping with this pathogen through therapeutics and vaccines.
